# Neurokinin 1 Receptor Mediates Membrane Blebbing and Sheer Stress-Induced Microparticle Formation in HEK293 Cells

**DOI:** 10.1371/journal.pone.0045322

**Published:** 2012-09-14

**Authors:** Panpan Chen, Steven D. Douglas, John Meshki, Florin Tuluc

**Affiliations:** 1 The Division of Allergy/Immunology, The Children’s Hospital of Philadelphia Research Institute, Philadelphia, Pennsylvania, United States of America; 2 The Department of Pediatrics, University of Pennsylvania, Pennsylvania, United States of America; 3 The Flow Cytometry Core Laboratory at The Children’s Hospital of Philadelphia Research Institute, Philadelphia, Pennsylvania, United States of America; University of Chicago, United States of America

## Abstract

Cell-derived microparticles participate in intercellular communication similar to the classical messenger systems of small and macro-molecules that bind to specialized membrane receptors. Microparticles have been implicated in the regulation of a variety of complex physiopathologic processes, such as thrombosis, the control of innate and adaptive immunity, and cancer. The neurokinin 1 receptor (NK1R) is a Gq-coupled receptor present on the membrane of a variety of tissues, including neurons in the central and peripheral nervous system, immune cells, endocrine and exocrine glands, and smooth muscle. The endogenous agonist of NK1R is the undecapeptide substance P (SP). We have previously described intracellular signaling mechanisms that regulate NK1R-mediated rapid cell shape changes in HEK293 cells and U373MG cells. In the present study, we show that the activation of NK1R in HEK293 cells, but not in U373MG cells, leads to formation of sheer-stress induced microparticles that stain positive with the membrane-selective fluorescent dye FM 2–10. SP-induced microparticle formation is independent of elevated intracellular calcium concentrations and activation of NK1R present on HEK293-derived microparticles triggers detectable calcium increase in SP-induced microparticles. The ROCK inhibitor Y27632 and the dynamin inhibitor dynasore inhibited membrane blebbing and microparticle formation in HEK293 cells, strongly suggesting that microparticle formation in this cell type is dependent on membrane blebbing.

## Introduction

The mechanisms of intercellular communication involve the release in the extracellular medium of messenger molecules that bind to receptors on target cells. Cells are also able to communicate via microvesicles, also known as microparticles, which are complex structures composed of a lipid bilayer with associated proteins that encloses a small part of cytoplasm from the donor cell. It has been described that microparticles affect other cells in various ways, from activating intracellular signaling pathways to transferring genetic material or proteins [1].

Cell-derived microparticles are heterogeneous and have diameters ranging from 50 to 2,000 nm [2], [3], [4]. Their formation is associated with three major cellular events: release of exosomes from late endosomes, cellular apoptotic breakdown, and membrane blebbing [5]. Exosomes are microvesicles with diameters smaller than 100 nm that are released from late endosomal compartments [2], [5]. Exosomes are found in supernatants of cultured cells and also in body fluids such as blood, urine, ascites and amniotic fluid [6]. A significant body of evidence supports the view that exosomes are a source of tumor antigens and they are involved in presentation of tumor antigens to T cells [7]. Exosomes also promote cell-to-cell spread of infectious agents, such as viruses and prions [8], [9]. Furthermore, exosomes isolated from cells infected with various intracellular pathogens, including *Mycobacterium tuberculosis* and *Toxoplasma gondii*, contain microbial components and can promote antigen presentation and macrophage activation, strongly suggesting that exosomes may function in immune surveillance [10], [11].

Microparticle formation due to apoptosis is generally a result of cell contraction leading to disruption of the cytoskeleton [12]. A variety of cells release microparticles during apoptosis, such as endothelial cells [13], [14], platelets [15], neutrophils [16], monocytes [17] and cancer cells [18], [19].

Membrane blebbing is often associated with apoptosis but it may also be an apoptosis-independent phenomenon triggered by activation of membrane receptors [20], [21]. During blebbing, microparticles may form as a result of bleb detachment from the cell bodies [16]. Work by Surprenant and colleagues demonstrated that activation of the P2X7 receptor leads to bleb formation, and that these blebs form microparticles that are released into the cellular environment [22].

The exact molecular signaling events required for the formation of different types of microparticles have not been clearly described. Lipid membrane redistribution is a characteristic feature of apoptosis and is regarded as essential for the release of microparticles from apoptotic cells [2], [23]. Upon cell death, phosphatidylserine, a phospholipid normally found on the cytosolic face of the plasma membrane, is translocated to the extracellular face of the membrane. This translocation is one of the major events required for triggering microparticle formation and release [23]. During microparticle formation in activated cells, intracellular calcium increase leads to changes in the activity of membrane proteins that control the localization of phosphatidylserine [2], [5], [23]. Exosome release occurs independently of phosphatidylserine exposure on the outer leaflet of the membrane [6].

The majority of blood microparticles originate from platelets (70–80%) [24], but monocytes, T cells, endothelial cells, and cancer cells are also able to generate microparticles [5], [24], [25]. Blood cell- and endothelium-derived microparticles are a major source of tissue factor and they participate in thrombus formation [24], [26], [27].

Microparticles are also important in the regulation of inflammatory responses, angiogenesis, nitric oxide production and regulation of the vascular tonus [25]. More recently it has been shown that microparticles can serve as a method of transfer of a variety of biologically active materials, such as oncoproteins, oncogenic DNA segments, oncogenic miRNA, oncogenic mRNA [5], [28], or retrotransposon RNA transcripts for endogenous retroviruses [29]. The cargo carried by microparticles can be transferred to nearby cells or to distant cells through the bloodstream and is capable of inducing biological changes that promote tumorigenesis, local invasion, and metastasis [30].

Neurokinin 1 receptor (NK1R) is a Gq-coupled receptor present in a variety of cells, including neurons in the central and peripheral nervous system, immune cells, cells in the endocrine and exocrine glands, smooth muscle cells, etc. [31]. Its endogenous agonist is substance P (SP), a neuropeptide that belongs to the tachykinin neuropeptide family. We have previously described intracellular signaling mechanisms that regulate NK1R-mediated rapid cell shape changes in HEK293 cells and U373MG cells [20], [21]. In the present study we show that the activation of the NK1R in HEK293 cells, but not in U373MG cells, leads to formation of microparticles. Because NK1R activation is associated with robust intracellular calcium increase, we have also investigated the ability of microparticles to respond with calcium increase to NK1R activation. Using inhibitors of key signaling molecules involved in membrane blebbing, [20], [21] we have developed experimental evidence that strongly suggests that microparticle formation in HEK293 cells is dependent on membrane blebbing.

## Materials and Methods

### Cells

The human astrocytoma cell line U373MG was purchased from American Type Culture Collection. HEK293 cells expressing the NK1R were obtained as previously described [32]. All cells were grown in DMEM supplemented with 10% fetal calf serum, glutamine, and antibiotics at 37°C in 5% CO_2_. HEK293 cells transfected with NK1R expressing Green Fluorescent Protein (GFP) were also grown as previously described [20]. The NK1R antagonist, aprepitant was purified by chromatography from Emend® capsules (Merck, Whitehouse Station, NJ) and solubilized in DMSO [32].

### Flow Cytometry

HEK293-NK1R cells cultured in 100 mm Petri dishes were detached and resuspended at a concentration of 3 million/ml. Cells were incubated for 45 minutes simultaneously with 5-chloromethylfluorescein diacetate (CFMDA; Invitrogen, Carlsbad, CA) in order to label the cytoplasm and with Nuclear-ID Red DNA stain (Enzo Life Sciences, Plymouth Meeting, PA) to label the nuclei, per manufacturers’ recommendations. After labeling, cells were washed and resuspended in Hank’s balanced salt solution (HBSS) containing 2 mM calcium chloride. Flow cytometry experiments were performed on an Accuri C6 analyzer (BD Biosciences, San Jose, CA) in the Flow Cytometry Core Laboratory of the Children’s Hospital of Philadelphia Research Institute. The blue laser (488 nm) was used for excitation; 530/30 nm bandpass and 670 nm longpass filters were used to detect the fluorescent light emitted by CFMDA and Nuclear-ID Red, respectively. Particles positive for CFMDA and Nuclear-ID Red were identified as intact cells, while microparticles were identified based on their variable green fluorescence intensity and lack of positive staining with Nuclear-ID Red. For each sample, 10,000 events corresponding to live cells were collected and the increase in number of microparticles was calculated in each sample. For the dose-dependent assay, cells were stimulated with various concentrations of SP (0.1 nM, 1 nM, 10 nM, and 100 nM) without pre-treatment with antagonists. The NK1R antagonist aprepitant was incubated with cells for 15 minutes prior to SP addition in order to block NK1R. For the time-dependent assay, the number of microparticles was determined in each sample before and at 3, 5, 10, and 15 minutes after addition of SP. Cell suspensions were mixed by pipeting prior to each flow cytometry analysis to develop shear stress that facilitates formation of microparticles. In preliminary experiments, we have determined that the mixing procedure does not trigger formation of microparticles if SP is not added to cells (data not shown). Y27632 and dynasore (Tocris Bioscience, Ellisville, MO) were used to inhibit ROCK and dynamin, respectively, by incubating HEK293-NK1R cells with the indicated concentrations of inhibitor at 37°C (for 20 and 30 minutes, respectively) prior to SP stimulation.

### Lipid Membrane Staining

Cells were incubated with the plasma membrane dye FM 2–10 per manufacturer’s instructions. Briefly, microparticles were generated by the addition of 100 nM SP then pelleted by centrifugation and resuspended in an ice-cold solution of FM 2–10 (5 µg/ml). Cells and microparticles were then immediately analyzed by flow cytometry as previously described to quantify median fluorescence intensity values (MFI) in stained cells compared to unstained control cells.

**Figure 1:**
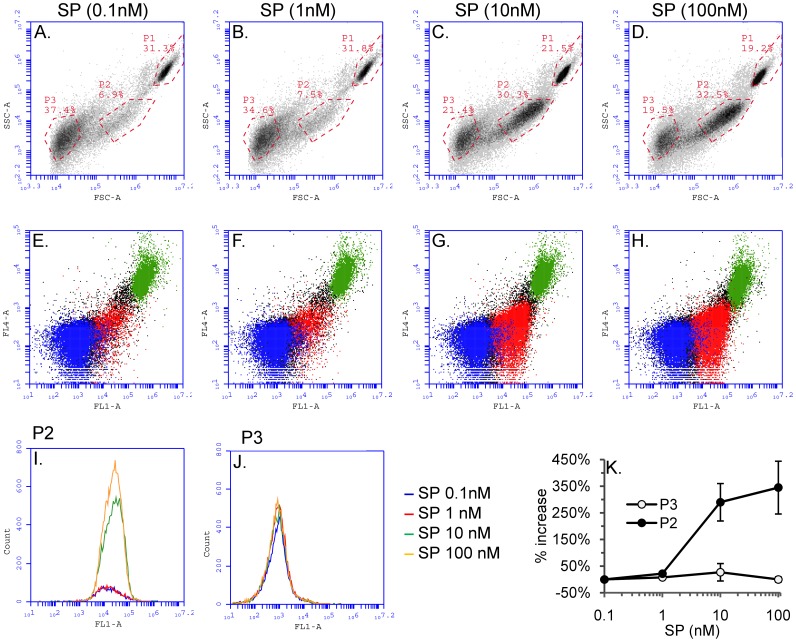
SP triggers sheer-stress induced microparticle formation in HEK293-NK1R cells in a dose-dependent manner. HEK293-NK1R cell suspensions were incubated for 10 minutes with different concentrations of SP as indicated and then analyzed by flow cytometry. *Panels (A–D)* Density plots showing intact cells (P1) and microparticles (P2 and P3) detected based on their light scattering properties: forward scatter (FSC-A) and side scatter (SSC-A). *Panels E–H)* Dot plots showing the staining pattern with the cytosolic dye CFMDA (FL1-A) and Nuclear-ID Red stain (FL4-A) in intact cells and microparticles. *Panels I and J)* Representative histograms showing the distribution of CFMDA fluorescence intensities (FL1-A) for P2 and P3 microparticles. *Panel K)* Line graph showing mean values ± SEM (n = 4) of microparticle counts expressed as percent increase as compared to baseline-stimulated cells. The number of microparticles in the P2 population significantly increased with the concentration on SP (p<0.05). No significant increase in the number of microparticles in the P3 population was found.

### Intracellular Calcium Measurements

Cells were incubated first with SP (100 nM) to induce microparticle formation, then microparticles were isolated from intact cells by centrifugation. Briefly, cell suspensions containing microparticles were centrifuged at 100 g for 5 minutes to pellet the intact cells. The microparticle suspension (“supernatant”) was removed and transferred to a new tube and then centrifuged at 1000 g for 5 minutes to pellet the microparticles. The pellets of intact cells and the microparticle pellets were washed, resuspended in HBSS containing 2 mM calcium chloride, and incubated for 1 h at 37°C to allow NK1R to resensitize. Intact cells and microparticles were then loaded in parallel with Fluo-4 AM (2 µM) and Fura-red AM (2 µM). Calcium recordings were performed using the Accuri C6 Analyzer. Fluorescence values were recorded in FL1-A (Fluo-4 AM) and FL3-A (Fura-red AM) channels. Intracellular calcium measurements were quantified by calculating the ratio between FL1-A/FL3-A values. Live cells and microparticles were both stimulated with 100 nM SP, ionomycin, or 1 µM ATP. The NK1R antagonist aprepitant (1 uM) was also used to examine the effects of blocking SP activation. For some experiments intact cells were not separated from microparticles before the second addition of SP, in order to allow for a direct comparison of responses in intact cells and microparticles. Cytosolic calcium measurements using the ratio of Fluo-4AM/Fura-red-AM fluorescence values has previously been used in other studies [33], [34]. The flow cytometry software package FlowJo (TreeStar Inc.) has been used for calculating derived parameters and for performing kinetic analysis of intracellular calcium responses.

### Confirmation of NK1R Presence on Microparticles

HEK293 cells were transfected with NK1R containing GFP as previously described [20]. HEK293-NK1R-GFP and non-fluorescent HEK293-NK1R cells were stimulated with SP to generate microparticles. Microparticles and live cells were isolated by centrifugation, and analyzed by flow cytometry to compare fluorescence values in NK1R-GFP cells and cells without GFP.

**Figure 2:**
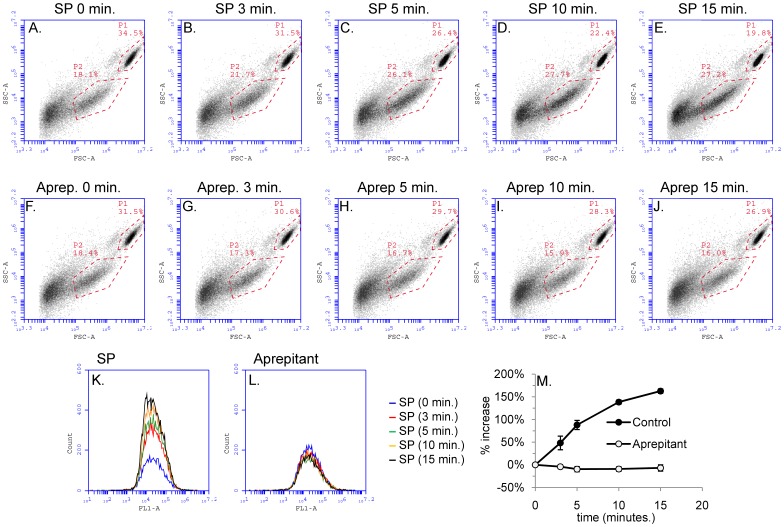
SP-induced microparticle formation in HEK293 cells is time-dependent. *Panels A–E*) Density plots showing forward scatter *vs.* side scatter properties of HEK293-NK1R cell suspensions treated with SP (100 nM). Repeated analyses were performed at various times after stimulation: A) prior to SP addition, B) 3 minutes, C) 5 minutes, D) 10 minutes, E) 15 minutes after addition of SP. The number of microparticles in the P2 population increased with time after addition of SP. *Panels F–J*) Density plots showing side vs. forward scatter properties of HEK293-NK1R cells pre-treated with NK1R antagonist aprepitant (1 µM) for 15 minutes prior to SP addition. *Panels K and L)* Representative histograms showing the distribution of CFMDA fluorescence intensities (FL1-A) for P2 microparticles. P2 microparticle formation increases with time after SP addition and is blocked by the treatment with aprepitant. *Panel M)* Line graph showing mean values ± SEM (n = 5) of microparticle count expressed as percent increase as compared to non-stimulated cells. Aprepitant blocks SP-induced formation of P2 microparticles.

### Staining of Endoplasmic Reticulum

HEK293-NK1R cells were incubated with the endoplasmic reticulum dye ER-Tracker Green (ER-TG) per manufacturer’s instructions. Briefly, a stock solution (1 mM) of ER-TG was added to cell suspended in HBSS containing calcium and magnesium and then cells were incubated for 20 minutes at standard culturing conditions (37°C, 5% CO_2_). After the incubation period was over, cells were washed twice in label-free HBSS, then microparticles were generated by SP activation. Cells and microparticles were then analyzed by flow cytometry to determine fluorescence values of stained versus unstained groups.

### Calcium Depletion

HEK293-NK1R cells were loaded with Fluo-4 AM (2 µM) and Fura-red AM (2 µM). Cells were then resuspended in calcium- and magnesium-free HBSS containing either EDTA (1 mM), EDTA (1 mM) and the intracellular calcium chelator BAPTA-AM (5 µM), or neither (control) and incubated for 20 minutes at room temperature, then stimulated with 100 nM SP. Intracellular calcium measurements were performed by flow cytometry. Flow cytometry was also used to determine microparticle count 10 minutes after SP stimulation, and for unstimulated cells.

**Figure 3:**
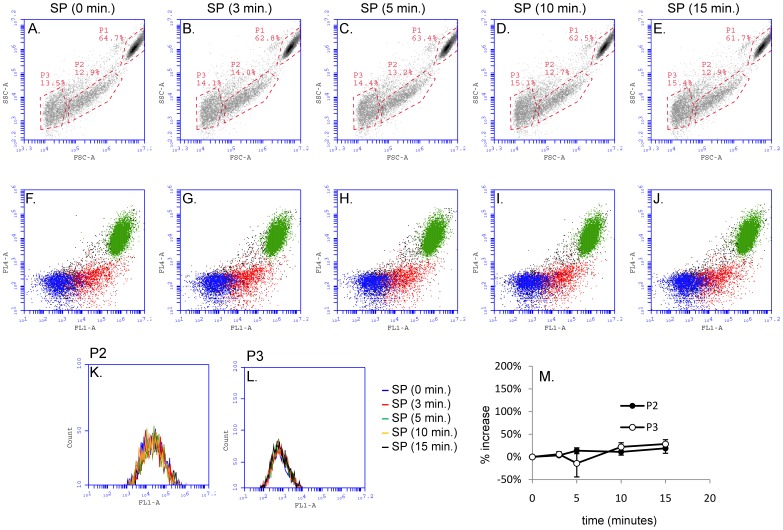
SP does not cause microparticle formation in U373MG cells. Cell suspensions of U373MG cells that express endogenous NK1R were incubated with SP (100 nM) for different intervals as indicated in each panel and then analyzed by flow cytometry. *Panels A–E)* Density plots showing intact cells (P1) and microparticles (P2 and P3) detected based on their light scattering properties. *Panels F–J)* Dot plots showing the staining pattern with the cytosolic dye CFMDA (Invitrogen; FL1-A) and Nuclear-ID Red stain (Enzo Life Sciences; FL4-A) in intact cells and microparticles. *Panels K and L)* Representative histograms showing the distribution of CFMDA fluorescence intensities (FL1-A) of P2 and P3 microparticles. *Panel M)* Line graph showing mean values ± SEM (n = 4) of microparticle counts expressed as percent increase as compared to non-stimulated cells. No significant increase in number of microparticles in the P2 and P3 populations was found.

### Membrane Blebbing Imaging

HEK293-NK1R cells were incubated with dynasore (100 µM) and Y27632 (10 µM) at 37°C for 20 minutes and 30 minutes, respectively. Phase-contrast images were taken with an IX51 Olympus microscope equipped with a Hamamatsu Orca C8484 camera. SP (100 nM) was added to media, then cells were imaged again to record cell morphology changes. Control cells (incubated with no inhibitor) were also stimulated with SP and imaged.

### Statistical Analysis

Three to six independent experiments were performed for each condition and mean values and standard errors were calculated. One way analysis of variance and the Student t-test were used to determine the statistical significance of differences between means (*p<0.05).

**Figure 4:**
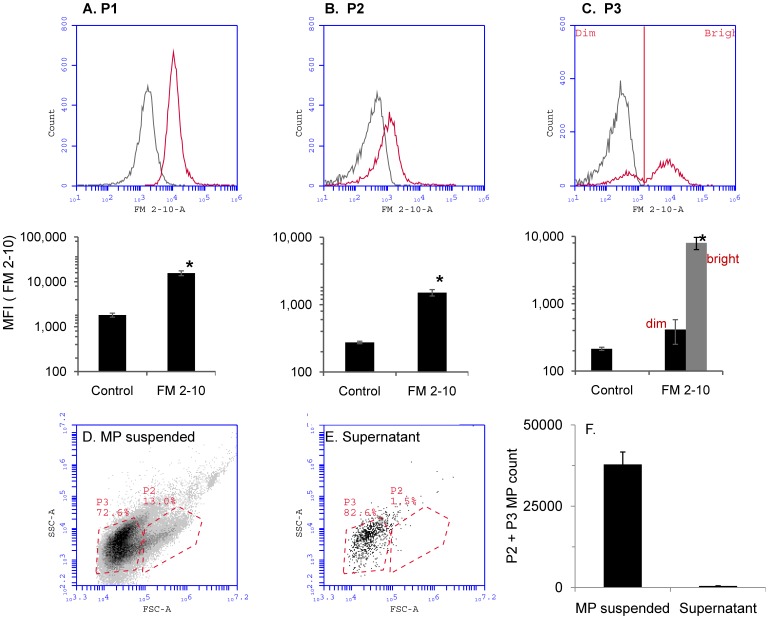
P2 and P3 microparticles originate from the plasma membrane. *Panels A–C)* Microparticles were generated by SP addition to HEK293-NK1R cells and stained with the plasma membrane dye FM 2-10 as described under *Materials and Methods*. Intact cells (A), P2 (B) and P3 (C) microparticles all stained positive, indicating that these vesicles contain membrane. *Upper panels* (grey curves: control cells or microparticles; red curves: stained cells or microparticles) and bar graphs (lower panels) showing median fluorescence values (MFI) for unstained and stained samples. A bimodal distribution histogram was found for the P3 population of microparticles (upper panel C) and the median fluorescence intensity was calculated separately for the dim and bright subpopulations of P3 microparticles (lower panel C). Data are derived from three independent experiments (mean values ± SEM; *p<0.05). *Panels D–F)* Microparticles were centrifuged at 10,000 g for 10 minutes. Both P2 and P3 microparticles were removed by centrifugation, indicating that these are not exosomes or protein aggregates.

## Results

In the present study we examined microparticle generation induced by SP in HEK293 cells expressing the NK1R receptor. We distinguished between intact cells and microparticles by flow cytometry, based on their forward and side scatter properties, as well as by using the cytosolic dye 5-chloromethylfluorescein diacetate (CFMDA) as a green fluorescent cytosolic marker, and Nuclear-ID Red stain as a nuclear marker. We used an Accuri C6 flow cytometer which can detect particles as small as 0.5 µm.

**Figure 5:**
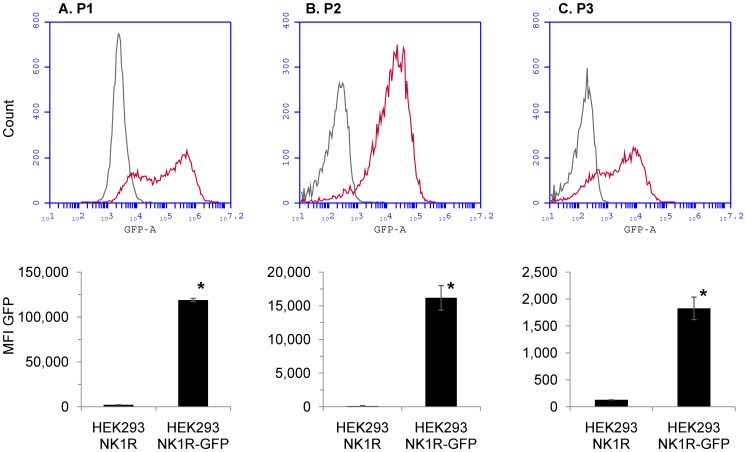
P2 microparticles contain NK1R. HEK293 cells were transfected with a plasmid encoding the NK1R-GFP fusion protein. HEK293-NK1R-GFP and HEK293-NK1R cells were stimulated with SP to generate microparticles, then analyzed by flow cytometry. *Upper panels:* histograms showing the distribution of GFP fluorescence in transfected cells, as compared to control cells. *Lower panels:* median fluorescence intensities obtained from three experiments. Intact cells (*upper panel A*), P2 (*upper panel B*) and P3 (*upper panel C*) microparticles exhibited higher fluorescence levels in samples transfected with GFP than in control samples transfected with untagged NK1R. Lower panels show median fluorescence intensities obtained from three experiments (mean values ± SEM; *p<0.05).

### SP Triggers Generation of Sheer-stress Induced Microparticles in HEK293 Cells in a Dose-dependent Manner

Two distinct populations of microparticles were detected by flow cytometry in HEK293 cells expressing NK1R, based on forward and side light scattering properties recorded, and also based on the data recorded in fluorescent channels after staining the cells with cytosolic and nuclear dyes. Cell suspensions were stimulated with different concentrations of SP (0.1 nM, 1 nM, 10 nM, and 100 nM) and flow cytometry analysis was performed 10 minutes after the addition of SP. In the density plots displayed in Fig. 1A*–*D intact cells (P1) and microparticles (P2 and P3) were identified based on their light scattering properties. The P1 population represents intact cells that have relatively high forward and side scatter values. P2 and P3 are two separate populations of microparticles. The P2 population has higher forward scatter values than P3, which indicates that P2 microparticles are larger than P3. In order to confirm the identity of the events detected, we loaded the cells with the green cytosolic dye CFMDA and with the DNA stain Nuclear-ID Red that labels the nuclei (Fig. 1E*–*H). The P1 population contained particles with high levels of staining for both CFMDA and Nuclear-ID Red, while the events in the P2 population were positive for the CFMDA stain, but negative for nuclear staining pattern. This staining pattern confirms that the particles in the P2 population contain small amounts of cytoplasm but no nuclei. The events in the P3 population did not stain positively with CFMDA, suggesting that these particles contain very small amounts of cytoplasm, if any, and they also did not stain with Nuclear-ID Red stain, indicating that they do not contain nuclei. To visualize changes in the distribution of CFMDA fluorescence, histograms were constructed for P2 and P3 populations of microparticles (representative results from one experiment are shown in Fig. 1I and 1J). The percentage increase in the number of P2 and P3 microparticles was calculated for each concentration of SP that was tested in three independent experiments. We have found that the mean percent increase of the number events in the P2 population was dependent on SP concentration, while the number of events in the P3 population did not increase with the concentration of SP (Fig. 1K). Because the generation of microparticles in the P3 populations was independent on NK1R activation, we focused our study on the microparticles in the P2 population.

**Figure 6:**
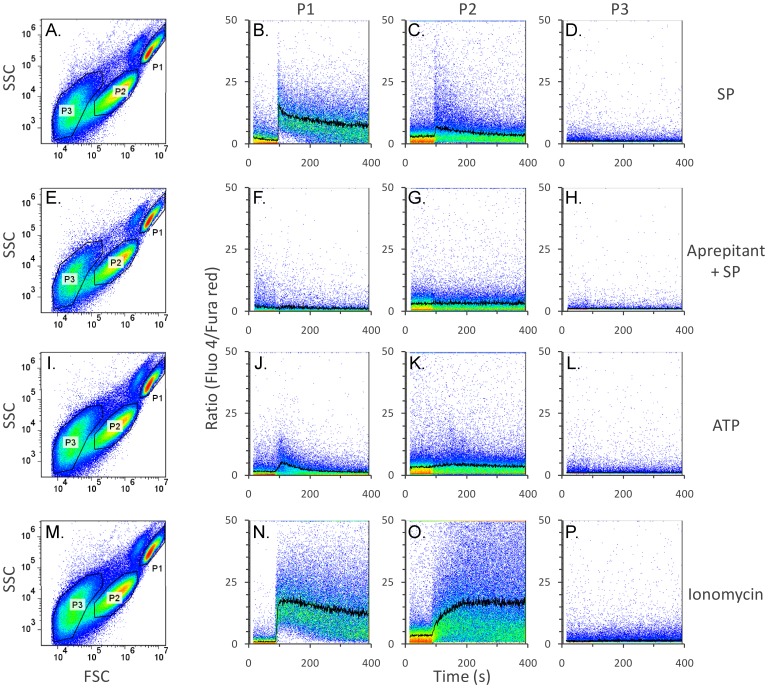
SP induces detectable intracellular calcium increase in P2 microparticles, but not in P3 microparticles. HEK293-NK1R cells were loaded with fluo-4 and fura red, as described under *Materials and Methods* and then stimulated with SP (100 nM) to generate microparticles. Microparticles and cells were washed to remove SP, and incubated for one hour at 37°C to allow NK1R to resensitize. SP (100 nM; panels A*–*H) was added to resensitized cell suspensions while performing intracellular calcium recordings. Aprepitant (1 µM) was used to block NK1R before addition of SP (panels E–H). ATP (1 µM; panels I*–*L), a P2 receptor agonist, and ionomycin (1 µM; panels M*–*P) were used to trigger intracellular calcium response independent of NK1R activation. *Panels A, E, I, M* show light scattering properties of representative samples. Line graphs of mean ratio (fluo-4/fura red) vs. time overlapped on pseudocolored dot plots ratio vs. time are shown in the remaining panels. *Panels A–D)* SP induced obvious intracellular calcium increase in intact cells and P2 microparticles, while P3 microparticles did not respond with detectable calcium increase to SP. *Panels E–H)* The NK1R antagonist aprepitant completely blocked the effect of SP, indicating that calcium increase is due to NK1R activation in intact cells as well as in P2 microparticles. Calcium increase induced by ATP (*Panels I–L)* and ionomycin (*Panels M–P*) are shown for comparison.

### SP Triggers Generation of Sheer-stress Induced Microparticles iin HEK293 Cells in a Time-dependent Manner

HEK293-NK1R cells loaded with CFMDA and Nuclear-ID Red stain were stimulated with SP (100 nM) and flow cytometry analyses were performed at 0, 3, 5, 10, and 15 minutes after SP stimulation (representative density plots are shown in Fig. 2A*–*E). We have found that the number of P2 microparticles increased with time after SP stimulation. We have also pre-treated HEK293-NK1R cells with the NK1R antagonist aprepitant (1 µM) for 15 minutes prior to addition of SP (100 nM) and flow cytometry recordings were performed at the same time intervals (representative density plots are shown in Fig. 2F*–*J). In cells treated with aprepitant, the number of P2 microparticles did not increase after SP addition. Representative histograms showing the distribution of green fluorescence (CFMDA) for the events recorded in the P2 population are presented in Fig. 2K and 2L. The mean values of percentage increase of the number of events in the P2 population obtained in four independent experiments are shown in Fig. 2M.

**Figure 7:**
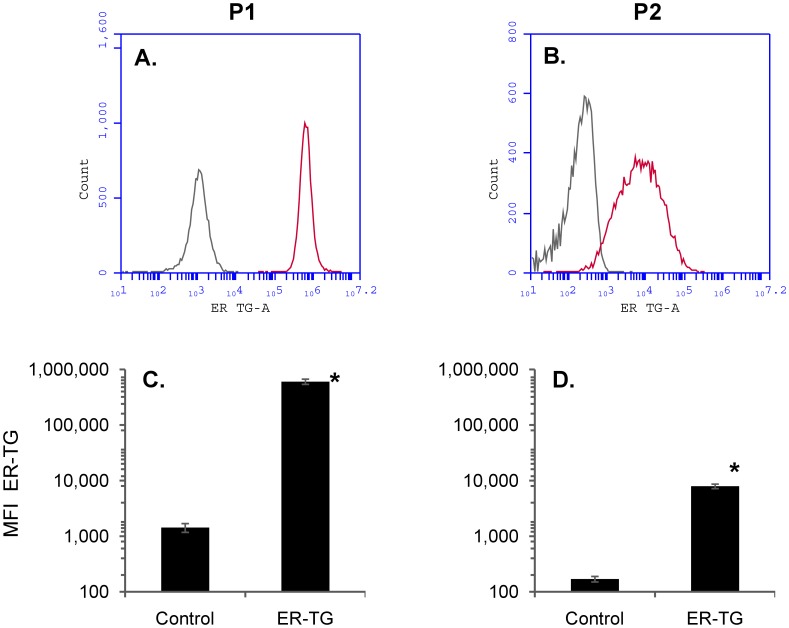
P2 microparticles contain endoplasmic reticulum. HEK293-NK1R cells were stained with the endoplasmic reticulum dye ER-TG and microparticles were generated by addition of SP. *Panels A and B*) Histograms depicting the distribution of the fluorescence intensities in cells and P2 microparticles in ER-TG-stained samples (*red curves*) and unstained samples (*grey curves*). *Panels C and D)* Mean fluorescence intensities obtained from three independent experiments (*p<0.05).

### Endogenous NK1R in U373MG Cells does not Mediate Formation of Microparticles

In order to determine if SP induces microparticle formation in other cell types, we used U373MG cells which endogenously express NK1R and respond to SP stimulation with cell morphology changes that are similar to those we observed in HEK293 cells expressing the recombinant NK1R [20]. A time course experiment was performed using the same experimental procedure described for HEK293-NK1R cells. Density plots for cells stimulated with SP are shown in Fig. 3A*–*E (forward *vs.* side scatter values) and dot plots showing the staining pattern with CFMDA and Nuclear-ID Red in intact cells and microparticles are shown in Fig. 3F*–*J (CFMDA, FL1-A *vs.* Nuclear-ID Red, FL4-A fluorescence values). Representative histograms showing the distribution of green fluorescence (CFMDA) for the events recorded in the P2 and P3 populations are presented in Fig. 3K and 3L. No significant changes in the number of microparticles in the P2 and P3 populations were detected (Fig. 3M).

**Figure 8:**
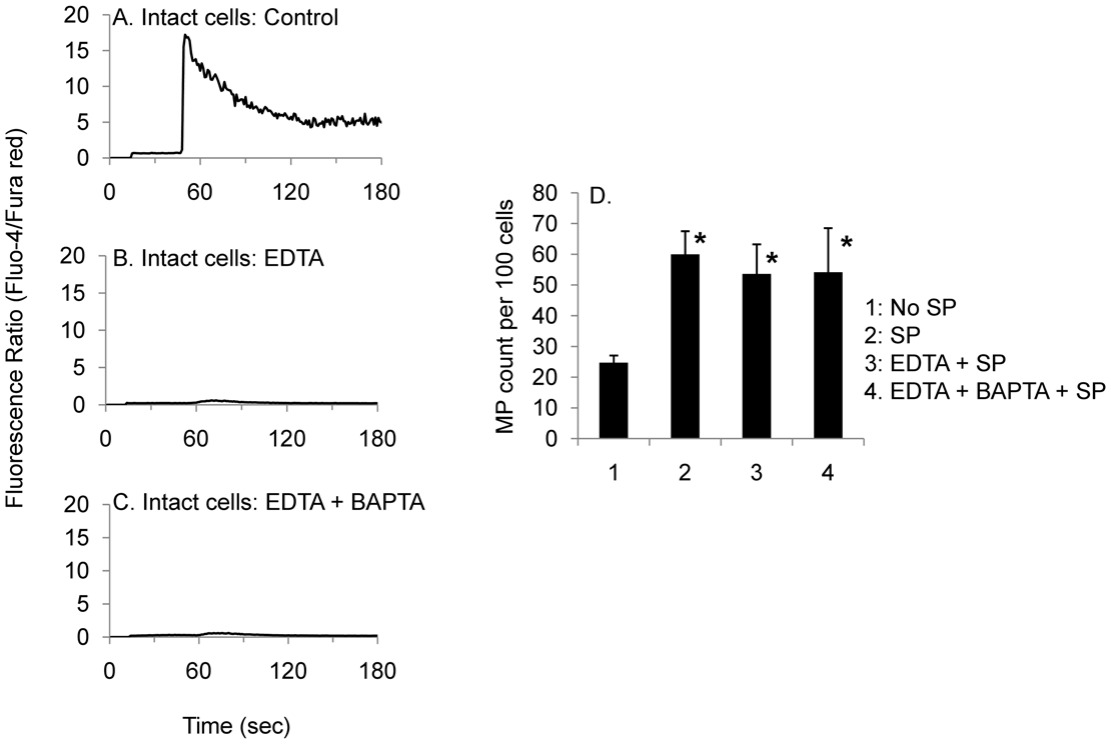
Microparticle formation is independent of intracellular calcium levels. *Panels A–C*: HEK293-NK1R cells were loaded with fluo-4 and fura red in (A) HBSS containing Ca^2+^/Mg^2+^, (B) Ca^2+^/Mg^2+^ free HBSS with EDTA, or (C) Ca^2+^/Mg^2+^ free HBSS with EDTA and BAPTA-AM. Cells were then stimulated with SP and intracellular calcium levels were measurement by flow cytometry. Cells incubated with EDTA and EDTA and BAPTA showed very low levels of intracellular calcium, indicating that calcium was effectively chelated and they responded to SP treatment with very small intracellular calcium increase. *Panel D)* Microparticles were counted by flow cytometry 10 minutes after addition of SP. The number of microparticles was relatively unaffected by EDTA and BAPTA, indicating that this response is independent of intracellular calcium levels.

### P2 and P3 Microparticles are not Exosomal Bodies and Originate from the Plasma Membrane

FM dyes are lipophilic styryl compounds widely used in studies involving cellular membranes and vesiculation. HEK293-NK1R cells were stained with the membrane dye FM 2–10. Both live cells and P2 microparticles stained positive (Figs. 4A,B), indicating that these vesicles originate from the plasma membrane. P3 microparticles however, had a bimodal distribution with a subpopulation brightly stained with FM 2–10 and a second population very dim (Fig. 4c). To determine if the microparticles (P3 particularly) were exosomes or simply protein aggregates, the microparticles were centrifuged at 10,000 g for 10 minutes. Both P2 and P3 microparticles were pelleted (Figs. 4D,E); the resulting supernatant exhibited far lesser numbers of microparticles (Fig. 4F).

### P2 Microparticles Contain NK1R

HEK293 cells were transfected with a plasmid encoding the NK1R-GFP fusion protein, as previously described [20]. HEK293-NK1R-GFP and HEK293-NK1R cells were stimulated with SP to generate microparticles, then analyzed by flow cytometry. As expected, higher fluorescence values were recorded for cells transfected with NK1R-GFP (Fig. 5A). The distribution of the fluorescence was bimodal, indicating that cells with different levels of GFP expression were present (Fig. 5). A similar bimodal aspect was found with the P3 population of microparticles (Fig. 5). However, the fluorescence distribution for the GFP-positive P2 microparticles was unimodal (Fig. 5B). In addition, we found higher fluorescence values in microparticles derived from HEK293-NK1R-GFP cells as compared to non-fluorescent cells, indicating that NK1R is present on the microparticle surface (Fig. 5B).

**Figure 9:**
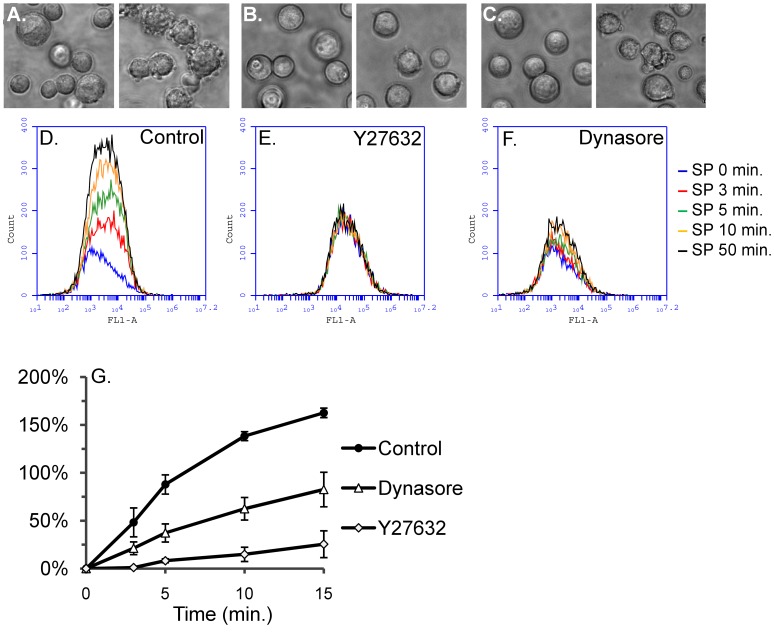
Signaling through ROCK and dynamin is required for membrane blebbing and SP-induced microparticle formation in HEK293-NK1R cells. HEK293-NK1R cells were incubated for 20 minutes with the ROCK inhibitor Y27632 (10 µM) or 30 minutes with the dynamin inhibitor dynasore (100 µM), then SP (100 nM) was added to media. Cells were monitored by phase contrast microscopy and microparticle formation was assessed by flow cytometry. *Panels A–C*) Phase contrast micrographs taken before (*left panels*) and after (*right panels*) addition of SP. *Panels D–F*) Representative histograms showing the distribution of CFMDA fluorescence intensities for P2 microparticles. *Panel G)* Line graph showing mean values ± SEM (n ≥3) of microparticle counts expressed as percent increase after SP stimulation as compared to non-stimulated cells.

### P2 Microparticles Respond with Cytosolic Calcium Increase when Stimulated with SP

To further determine if microparticles generated as a consequence of SP stimulation contain functional NK1R and are able to mediate intracellular calcium responses, we used a ratiometric calcium assay. Cells were first stimulated with SP (100 nM) to generate microparticles, then cells and microparticles were washed to remove SP, and kept for 1 hour at 37°C, in an atmosphere containing 5% carbon dioxide to allow the NK1R to resensitize. Separated microparticles and cells were loaded with Fluo-4 and Fura-red, then stimulated again with 100 nM SP and intracellular calcium measurements were performed. Density plots showing forward vs. side scatter values for cells and microparticles are shown in Fig. 6A, E, I and M. Intact cells responded with robust intracellular calcium increase (Fig. 6B). P2 microparticles had relatively high basal levels of calcium as compared to intact cells and many of them responded with large intracellular calcium increase to SP stimulation (Fig. 6C). P3 microparticles did not respond to SP addition with calcium increase (Fig. 6D). In a separate experiment, intact cells and microparticles were incubated with the NK1R antagonist aprepitant (1 µM), then stimulated with SP. Figs. 6F*–*H show that aprepitant inhibits the calcium response in both live cells and P2 microparticles. We also stimulated live cells and P2 microparticles with ATP (1 µM) and ionomycin (1 µM), both resulting in calcium increases (Fig. 6).

### P2 Microparticles Contain Endoplasmic Reticulum

Endoplasmic reticulum plays a critical role in generating intracellular calcium signals in most mammalian cells. To determine if endoplasmic reticulum is present in P2 microparticles that respond with calcium increase to SP activation of HEK293-NK1R receptor, cells were incubated with the endoplasmic reticulum dye ER-Tracker Green. Both live cells and P2 microparticles stained positive, indicating that microparticles contain ER (Fig. 7).

### Microparticle Formation is Independent of Intracellular Calcium Levels

We have previously shown that although NK1R is best known as a Gq-coupled receptor that signals through intracellular calcium increase, membrane blebbing induced by SP is a calcium-independent phenomenon [21]. To demonstrate that microparticle formation is also calcium-independent, cells were incubated with either EDTA or EDTA and BAPTA. Flow cytometry was used to quantify intracellular calcium changes and microparticle formation. Addition of EDTA or the combination of EDTA and BAPTA blocked calcium increases in live cells (Figs. 8A*–*C). However, inhibition of calcium increase did not have a significant effect on microparticle formation (Fig. 8D).

### ROCK and Dynamin Activity are Required for NK1R-mediated Membrane Blebbing and Microparticle Formation

To determine if membrane blebbing is a prerequisite for NK1R-mediated microparticle formation we have used several compounds that interfere with membrane blebbing through different mechanisms. We used the ROCK inhibitor Y27632 and the dynamin inhibitor dynasore. HEK293-NK1R cells were treated separately with Y27632 (10 µM) or dynasore (100 µM), or received no treatment (control). Flow cytometry recordings were performed in order to assess microparticle generation and phase contrast micrographs (Fig. 9A*–*C) were taken to visualize cell morphology before and after addition of SP (100 nM). Histograms were constructed based on CFMDA green fluorescence to show distribution of microparticles (Fig. 9D*–*F). The mean values obtained in all experiments (n≥3) were calculated as percent increase in microparticle count as compared to non-stimulated cells (Fig. 9G). SP-induced membrane blebbing (visible in Fig. 9A, right panel) and microparticle formation were inhibited in cells treated with Y27632 and dynasore.

## Discussion

When stimulated with SP, HEK293-NK1R cells generate two populations of microparticles, which are referred to as P2 and P3. The formation of microparticles belonging to the P3 population was not responsive to increases in SP concentration, indicating that this response is not due to SP. We concluded that cells release this type of microvesicles independently of SP stimulation. As shown in Fig. 1A, there is a basal level of P2 and P3 microparticles present without SP stimulation. However, addition of SP does not increase the basal level of P3 microparticles. We also note that the Accuri C6 analyzer can only detect particles greater than 0.5 µM, so we were unable to characterize particles smaller than those in the P3 population. In contrast, the number of microparticles in the P2 populations increased after adding SP to media in a dose- and time-dependent manner (Figs. 1 and 2), strongly suggesting that this event was mediated by NK1R, the receptor for substance P. Treatment of cells with the NK1R antagonist aprepitant completely blocked P2 microparticle formation (Fig. 2), proving that this response is mediated by NK1R.

We have recently shown SP induces similar morphology changes in U373MG glioblastoma cells that express endogenous NK1R and in HEK293 cells transfected with the recombinant NK1R [20]. Despite the similarities observed, we found that the intracellular signaling mechanisms activated downstream of NK1R in U373MG *vs.* HEK293 cells are distinct, especially with respect to the phosphorylation of p21-activated kinase, which occurs in U373MG but not in HE293-NK1R cells. SP-induced membrane protrusions in HEK293 cells are larger and longer lasting than in U373 cells. We now show that the larger blebs in HEK293 cells are associated with SP-induced microparticle formation, while the smaller and more transient blebs observed in U373MG cells are not associated with formation of microparticles (Fig. 3). We have observed that small levels of shear stress generated by mixing the cells by repeated pipeting are needed for generating microparticles in HEK293 cells. In the absence of shear stress the membrane protrusions remain attached to cell bodies and eventually end up forming new adhesions with the neighboring cells or with the bottom of the cell culture dish [20].

The data obtained with the membrane dye FM 2–10 strongly suggests that P2 microparticles released from HEK293-NK1R cells are derived from the plasma membrane (Fig. 4), since the fluorescence intensity of stained microparticles was clearly shifted to the right (Fig. 4B). Interestingly, we have found that only approximately 51% of the P3 microparticles stain positive with FM 2–10, suggesting that not all of them have lipids similar to intact cells. A representative histogram is shown in Fig. 4C. All microparticles were pelleted at a relatively low speed of 10,000 g indicating that they are not protein aggregates or exosomal bodies, which require much higher speeds of 100,000 g or more to pellet.

In order to demonstrate that NK1R is present on microparticles, HEK293 cells were transfected with NK1R fused to GFP. We have found that after stimulation with SP the microparticles generated from these cells had increased levels of fluorescence, as compared to non-fluorescent HEK293-NK1R cells, indicating that they contain NK1R. Given the paramount roles of G protein-coupled receptors in cellular signaling, the presence of NK1R on microparticles suggests that microparticle release may be a form of intercellular communication. It is conceivable that G protein-coupled receptors on microparticles derived from cancer cells may trigger biological responses with important outcomes, such as for the release of oncogenic materials, thus contributing to tumor growth and metastasis (Rak 2010). Due to variable expression levels of NK1R-GFP expression in transfected cells, the histogram in Fig. 5A has a bimodal distribution. Interestingly, although the bimodal distribution is also observed in the P3 population of microparticles, we have consistently found that the microparticles in the P2 population had a unimodal distribution. The implications of this observation are not clear, but it is possible that an optimal density of NK1R is required on the membrane of the cell in order to generate microparticles as a consequence of SP activation. Additional investigations will be required in order to test this hypothesis.

Activation of functional NK1R leads to intracellular calcium increase due to release of calcium ions from the endoplasmic reticulum and to calcium influx from the extracellular environment. To further determine if NK1R receptors are functional on the surface of microparticles, we measured the intracellular calcium increase induced by SP in microparticles and intact cells. We have shown that SP stimulation causes modest calcium increase in the P2 population of microparticles (Fig. 6B), clearly indicating that these microvesicles contain functional NK1R. The response was found to be statistically significant, with a p-value of 0.032. We have observed great variability in the responses induced in P2 microparticles and although the mean values of the ratio Fluo-4/fura read did not indicate very robust increases, analysis of individual microparticles showed that many of them respond with calcium increase as high as in intact cells. We have also observed large calcium increase induced by ionomycin in intact cells and P2 microparticles (Fig. 6N, O).

HEK293 cells endogenously express purinergic receptors that are activated by extracellular nucleotides, such as ATP. We show that ATP causes an obvious intracellular calcium increase in intact cells (Fig. 6J), while the response to ATP in microparticles is barely detectable (Fig. 6K).

We were not able to trigger calcium response in P3 microparticles, strongly suggesting that this type of particles contains non-functional receptors.

The cause of higher baseline levels of intracellular calcium in non-stimulated microparticles may also be due to the fact that when the microparticles separate from the cell, a breach in the integrity of the plasma membrane is likely to form, causing extracellular calcium from the medium to leak into the microparticles. In a separate experiment, cells were treated with EDTA to chelate extracellular calcium. This treatment caused, as expected, very low baseline levels of intracellular calcium in non-stimulated microparticles but also resulted in complete loss of the response to SP (data not shown), possibly due to the fact that calcium chelation led to depletion of calcium from microparticles.

The source of calcium influx in microparticles may partially originate from the ER. Using the ER-Tracker green dye we have shown that P2 microparticles contain ER (Fig. 7). Thus, it seems that P2 microparticles maintain a complete molecular equipment for triggering cell-like responses. P3 microparticles did not respond with calcium increase and we have found that they stained dimly with ER-Tracker green, suggesting that although some of them retain ER, the amount may not be sufficient to sustain measurable calcium release.

We have also determined that microparticle formation is independent of intracellular calcium levels. When cells were incubated with EDTA or EDTA and BAPTA, we found an almost complete inhibition of calcium increase (Fig. 8). However, there was not a significant decrease in microparticle formation, indicating that this response is not dependent on cytosolic calcium levels.

The Rho/ROCK signaling pathway is involved in NK1R-mediated membrane blebbing [20], [21]. In the present study we show that cells treated with the ROCK inhibitor were unable to form microparticles. The enzyme dynamin is well-recognized as a key molecule implicated in clathrin-coated vesicle budding during endocytosis [35], [36]. Membrane remodeling via endosomal recycling through endo- and exocytosis mechanisms is thought to be required for major changes of the cell shape during various cellular processes (e.g. mitosis) [37], [38]. Dynamin may be indirectly implicated in the regulation of membrane blebbing. In a recent study, dynasore increased the persistence of blebbing in freshly detached cells, possibly due to its inhibitory effect on endocytosis [38]. Because our data supports the hypothesis that microparticle formation in HEK293-NK1R cells is dependent on membrane blebbing, we investigated the role of dynamin in HEK293-NK1R cells. In the present study we bring evidence supporting the view that dynamin is required for membrane blebbing and microparticle formation. In cells treated with the dynamin inhibitor dynasore, we observed inhibition, albeit incomplete, of both membrane blebbing and microparticle formation (Fig. 9). Therefore, we suggest that dynamin regulates blebbing and microparticle formation through a mechanism distinct than ones previously described in other studies [38].

We have shown that HEK293-NK1R cells form at least two distinct types microparticles upon SP stimulation. Furthermore, we have shown the microparticles induced by SP respond with calcium increase to SP, indicating that these microparticles contain functional NK1R. We have also shown that the generation of microparticles is a calcium-independent process and we bring evidence supporting an essential role of Rho/ROCK and dynamin signaling functions. These proteins are also required for membrane blebbing, indicating that membrane blebbing is a major cellular event that induces microparticle formation.
